# Supplementary material for: Analysis of PPARγ Signaling Activity in Psoriasis
Source: Int J Mol Sci. 2021 Aug 10;22(16):8603. doi: 10.3390/ijms22168603 (PMC8395241; doi:10.3390/ijms22168603)
Supplement: Supplementary file 1 [file ijms-22-08603-s001.zip › Supplemental materials_Analysis of PPARg signaling activity in psoriasis/Pathway models/Models images and html files/Model of PPARG related pathways in psoriasis (short version)/10235.html]

S100A12


# Protein S100A12

|  |  |
| --- | --- |
| URN | urn:agi-llid:6283 |
| Total Entities | 0 |
| Connectivity | 762 |
| Name | S100A12 |
| Description | S100 calcium binding protein A12 |
| Notes | The protein encoded by this gene is a member of the S100 family of proteins containing 2 EF-hand calcium-binding motifs. S100 proteins are localized in the cytoplasm and/or nucleus of a wide range of cells, and involved in the regulation of a number of cellular processes such as cell cycle progression and differentiation. S100 genes include at least 13 members which are located as a cluster on chromosome 1q21. This protein is proposed to be involved in specific calcium-dependent signal transduction pathways and its regulatory effect on cytoskeletal components may modulate various neutrophil activities. The protein includes an antimicrobial peptide which has antibacterial activity. [provided by RefSeq, Nov 2014] |
| ObjectType | Protein |

---

|  |  |
| --- | --- |
| Pathway | Toll-like Receptor Independent Sterile Inflammation |
|  | Proteins Involved in Atherosclerosis |
|  | RAGE/AGER and S100 Proteins in Cardiovascular Injury |
|  | Advanced Glycation End Products Pathway in Diabetic Microangiopathy |
|  | Proteins Involved in Inflammatory Bowel Diseases |
|  | Proteins Involved in Endometriosis |
|  | Alveolar Macrophages Dysfunction in COPD |
|  | T-Cell Cytotoxic Activation |
|  | Alveolar Epithelial Cell Dysfunction in COPD |
|  | pprarg neg, uknown targets, ps-positive |
|  | prarg neg,ukn expres targets, ps-positive |
|  | PPARG negative targets |
|  | pparg expr target\_regulators |
|  | Neighbors of disease exacerbation |
|  | Neighbors of mortality |
|  | Neighbors of tumor recurrence |
|  | Neighbors of adverse outcome |
|  | Neighbors of leukocyte count |
|  | Neighbors of disease exacerbation |
|  | Neighbors of mortality |
|  | Neighbors of tumor recurrence |
|  | Neighbors of adverse outcome |
|  | Neighbors of leukocyte count |
|  | Neighbors of immune response |
|  | Neighbors of inflammatory response |
|  | Neighbors of wound healing |
|  | Neighbors of innate immune response |
|  | Neighbors of cell function |
|  | Neighbors of adaptive immune response |
|  | Neighbors of immunity |
|  | Neighbors of chemotaxis |
|  | Neighbors of cell survival |
|  | Neighbors of osteoclast differentiation |
|  | Neighbors of cell phenotype |
|  | Neighbors of cell adhesion |
|  | Neighbors of cell count |
|  | Neighbors of cell infiltration |
|  | Neighbors of SMC proliferation |
|  | Neighbors of cell damage |
|  | Neighbors of bone resorption |
|  | Neighbors of monocyte migration |
|  | Neighbors of neutrophil recruitment |
|  | Neighbors of cell migration |
|  | Neighbors of leukocyte migration |
|  | Neighbors of leukocyte recruitment |
|  | Neighbors of monocyte recruitment |
|  | Neighbors of ossification |
|  | Neighbors of cell growth |
|  | Neighbors of ROS generation |
|  | Neighbors of cell differentiation |
|  | Neighbors of cell homeostasis |
|  | Neighbors of psoriasis |
|  | Neighbors of inflammatory disease |
|  | Neighbors of atherosclerosis |
|  | Neighbors of colitis |
|  | Neighbors of arthritis |
|  | Neighbors of inflammatory bowel disease |
|  | Neighbors of atherogenesis |
|  | Neighbors of vascular remodeling |
|  | Neighbors of metastasis |
|  | Neighbors of carcinogenesis |
|  | Neighbors of chronic inflammation |
|  | Neighbors of inflammation |
|  | Neighbors of pathological angiogenesis |
|  | Neighbors of vascular disease |
|  | Neighbors of diabetes mellitus |
|  | Neighbors of asthma |
|  | Neighbors of pneumonia |
|  | Neighbors of osteoarthritis |
|  | Neighbors of chronic obstructive pulmonary disease |
|  | Neighbors of infection |
|  | Neighbors of preeclampsia |
|  | Neighbors of sepsis |
|  | Neighbors of autoimmune disease |
|  | Neighbors of ischemic stroke |
|  | Neighbors of neutrophil infiltration |
|  | Neighbors of lung injury |
|  | Neighbors of acute lung injury |
|  | Neighbors of gastric cancer |
|  | Neighbors of atherosclerotic plaque |
|  | Neighbors of lung disease |
|  | Neighbors of cardiovascular disease |
|  | Neighbors of type 2 diabetes |
|  | Neighbors of neoplasm |
|  | Neighbors of endometriosis |
|  | Neighbors of vasculitis |
|  | Neighbors of coronary artery disease |
|  | Neighbors of osteolysis |
|  | Neighbors of epithelial cell |
|  | Neighbors of keratinocyte |
|  | Neighbors of vascular smooth muscle cell |
|  | Neighbors of monocyte |
|  | Neighbors of macrophage |
|  | Neighbors of activated monocyte |
|  | Neighbors of fibroblast |
|  | Neighbors of endometrium cell |
|  | Neighbors of mononuclear cell |
|  | Neighbors of inflammatory cell |
|  | Neighbors of neutrophil |
|  | Neighbors of mononuclear phagocyte |
|  | Neighbors of granulocyte |
|  | Neighbors of myeloid cell |
|  | Neighbors of leukocyte |
|  | Neighbors of T-cell |
|  | Neighbors of phagocyte |
|  | Neighbors of intestine |
|  | Neighbors of aorta |
|  | Neighbors of brain |
|  | Neighbors of cardiovascular system |
|  | Neighbors of cornea |
|  | Neighbors of ileum |
|  | Neighbors of NF-kB family |
|  | Neighbors of Jun/Fos |
|  | Neighbors of PKC |
|  | Neighbors of PI3K |
|  | Neighbors of TLR |
|  | Neighbors of cytokine |
|  | Neighbors of interferon |
|  | Neighbors of JAK |
|  | Neighbors of NAD(P)H oxidase |
|  | Neighbors of TLR1/2 heterodimer |
|  | Model of PPARG signaling in psoriasis |
|  | PPARG negative regulators and targets |
|  | Model of PPARG related pathways in psoriasis (short version) |
|  | New Pathway (5) |
|  | dsRNA induces innate immunity and INFs in alveolar cells |
|  | AGER feed forward loop in innate immunity |
|  | 3\_Arterial wall inflammation and calcification in atherosclerosis |
|  | 1\_Cigarette smoke causes dysfunction and production of inflammatory mediators in alveolar epithelial cells |
|  | 3\_Role of alveolar macrophages in pulmonary tissue destruction in COPD |
|  | Model of PPARG signaling in psoriais (tested) |

---

|  |  |
| --- | --- |
| MedScan ID | 6283 |

---

|  |  |
| --- | --- |
| LocusLink ID | 6283 |

---

|  |  |
| --- | --- |
| Alias | CAAF I |
|  | CAAF1 |
|  | S100 calcium binding protein A12 |
|  | extracellular newly identified RAGE-binding protein |
|  | Calcium- binding protein in amniotic fluid 1 |
|  | S100 calcium binding protein A12 (calgranulin C) |
|  | Calgranulin C |
|  | S100A12 |
|  | Neutrophil S100 protein |
|  | EN-RAGE |
|  | migration inhibitory factor-related protein 6 |
|  | CAGC |
|  | OTTHUMP00000015332 |
|  | Calcium- binding protein in amniotic fluid I |
|  | calcitermin |
|  | ENRAGE |
|  | p6 |
|  | CGRP |
|  | MRP6 |
|  | MRP-6 |
|  | calcium-binding protein in amniotic fluid 1 |
|  | protein S100-A12 |
|  | S100 calcium-binding protein A12 (calgranulin C) |
|  | calgranulin-C |

---

|  |  |
| --- | --- |
| GO ID | 0050786 |
|  | 0005509 |
|  | 0005507 |
|  | 0008270 |
|  | 0061844 |
|  | 0050663 |
|  | 0042742 |
|  | 0050832 |
|  | 0006954 |
|  | 0045087 |
|  | 0031640 |
|  | 0045576 |
|  | 0002548 |
|  | 0030593 |
|  | 0043312 |
|  | 0043123 |
|  | 0043406 |
|  | 0051092 |
|  | 0050729 |
|  | 0006805 |
|  | 0005737 |
|  | 0005856 |
|  | 0005829 |
|  | 0005576 |
|  | 0005634 |
|  | 0005886 |
|  | 0034774 |
|  | 0005515 |

---

|  |  |
| --- | --- |
| KEGG ID | hsa:6283 |

---

|  |  |
| --- | --- |
| Organism | Homo sapiens {Organism urn:agi-taxid:9606} |
|  | Homo sapiens |

---

|  |  |
| --- | --- |
| OMIM ID | 603112 |

---

|  |  |
| --- | --- |
| Hugo ID | 10489 |
|  | HGNC:10489 |

---

|  |  |
| --- | --- |
| Human chromosome position | 1q21.3 |
|  | 1q21 |

---

|  |  |
| --- | --- |
| Swiss-Prot Accession | P80511 |
|  | P80511.2 |
|  | P83219 |
|  | Q5SY66 |
|  | Q7M4R1 |

---

|  |  |
| --- | --- |
| PIR ID | A61522 |
|  | JC4712 |

---

|  |  |
| --- | --- |
| GenBank ID | NC\_000001 |
|  | NM\_005621 |
|  | NP\_005612 |
|  | AL591704 |
|  | CH471121 |
|  | EAW53332 |
|  | D83657 |
|  | BAA12030 |
|  | X98288 |
|  | X98289 |
|  | CAB94792 |
|  | X98290 |
|  | BC070294 |
|  | AAH70294 |
|  | D49549 |
|  | BAA08497 |
|  | D83664 |
|  | BAA12036 |
|  | X97859 |
|  | CAA66453 |
|  | P80511 |
|  | NC\_018912 |
|  | ADR82637 |
|  | CAI19495 |
|  | HQ257882 |
|  | AMYH02001902 |
|  | ABBA01049433 |
|  | AC\_000133 |

---

|  |  |
| --- | --- |
| Swiss-Prot ID | S10AC\_HUMAN |

---

|  |  |
| --- | --- |
| Cell Localization | Secreted |
|  | Cytoplasm |
|  | Cytoskeleton |
|  | Cell membrane |
|  | Cytoplasm, cytoskeleton |

---

|  |  |
| --- | --- |
| Ensembl ID | ENSG00000163221 |
|  | ENSP00000357726.3 |
|  | ENST00000368737.5 |
|  | ENSP00000357726 |
|  | ENST00000368737.4 |
|  | ENST00000368737 |

---

|  |  |
| --- | --- |
| Unigene ID | Hs.19413 |

---

|  |  |
| --- | --- |
| IPI ID | IPI00218131 |

---
